# Anomalous Origin of the Right Coronary Artery Causing Myocardial Ischemia: A Case for a Multimodality Imaging Approach

**DOI:** 10.1155/2021/6686227

**Published:** 2021-03-19

**Authors:** Fatimah A. Alkhunaizi, Karan Kapoor, Vincent Pallazola, Edward P. Shapiro, Peter V. Johnston, Joban Vaishnav, Nisha A. Gilotra, Ahmet Kilic, Rosanne Rouf

**Affiliations:** ^1^Department of Medicine, Johns Hopkins Hospital, Baltimore, MD, USA; ^2^Division of Cardiology, Department of Medicine, Johns Hopkins Hospital, Baltimore, MD, USA; ^3^Division of Cardiothoracic Surgery, Department of Surgery, Johns Hopkins Hospital, Baltimore, MD, USA

## Abstract

A 46-year-old man was admitted with non-ST elevation myocardial infarction and newly diagnosed acutely decompensated heart failure. Echocardiogram demonstrated left ventricular ejection fraction of 30% with basal inferior and inferolateral akinesis. Coronary angiography showed mild diffuse coronary artery disease and an anomalous right coronary artery arising from the left coronary cusp. Further imaging was consistent with ischemia in the right coronary distribution. Etiology of ischemia was thought to be the anomalous right coronary artery, and surgical unroofing of the right coronary ostium was performed. Here, we report a multimodality imaging approach, including cardiac magnetic resonance, cardiac computed tomographic angiography, and single-photon emission computed tomography, to support the diagnosis and management of a patient with anomalous right coronary artery arising from the left coronary cusp.

## 1. History of Presentation

A 46-year-old man from Honduras with no prior medical history presented with two weeks of crescendo angina, exertional dyspnea, two-pillow orthopnea, and paroxysmal nocturnal dyspnea. He took no medications and had no history of alcohol or illicit substance use. He had no family history of cardiovascular disease. He reported an active childhood and adolescence and was employed in a labor-intensive profession installing fiber optic cables for a telecommunication company. Physical examination was notable for a blood pressure of 178/81 mmHg, heart rate of 100 beats/min, a third heart sound, and mild jugular venous distention. The lung fields were clear, and there was no peripheral edema. Resting electrocardiogram showed sinus tachycardia and flattened T-waves in leads II, III, aVF, V5, and V6. Troponin-I was initially 0.35 ng/mL, peaking at 7.16 ng/mL upon serial assessment. Pro-BNP was elevated at 3961 pg/mL.

## 2. Investigations

**Figure 1:**
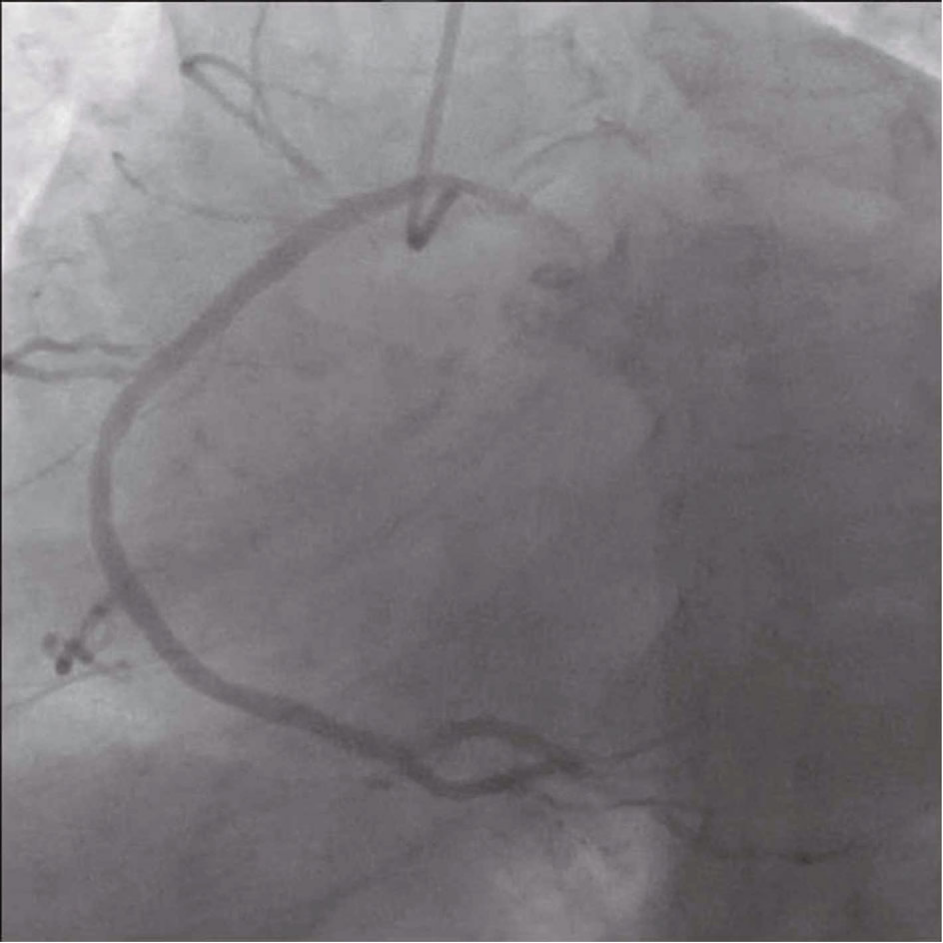
Still frame image of the RCA in the right anterior oblique (RAO) projection. The RCA is seen arising from the left coronary cusp prior to taking a usual course through the atrioventricular groove.

**Figure 2:**
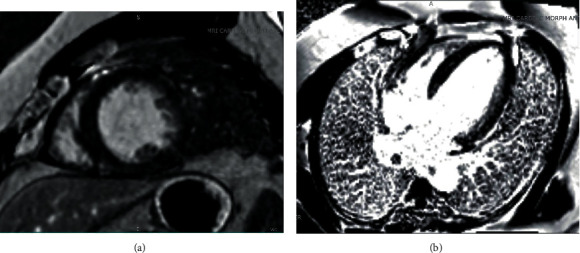
(a, b) Short-axis and four-chamber myocardial delayed enhancement imaging performed 10 minutes following injection of gadolinium contrast demonstrated no evidence of late gadolinium enhancement.

**Figure 3:**
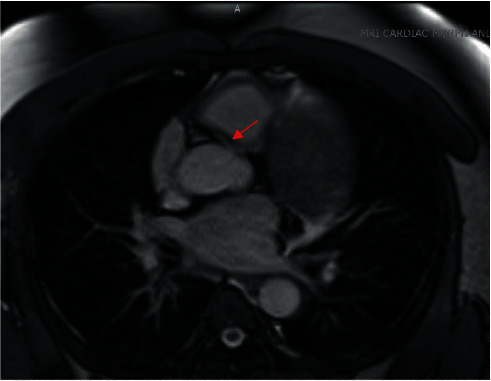
Axial CMR images corroborating the anomalous RCA (arrow) arising from the left coronary cusp and appearing to take an intra-arterial course.

**Figure 4:**
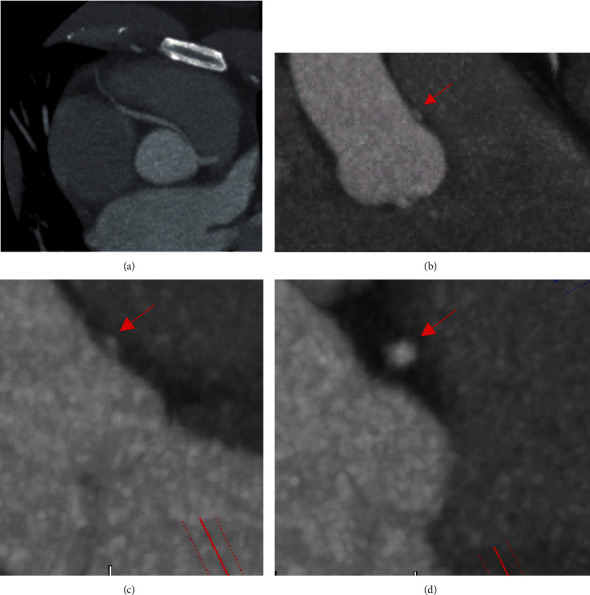
(a–d) Axial CCTA images demonstrating the anomalous RCA arising from the left coronary cusp and taking an intra-arterial course. The extent of the proximal and mid-vessel is better delineated than in CMR (a). The slit-like configuration of the ostial RCA, as well as the acute angulation of its takeoff, is seen in this en-face coronal view (b). Cross sections of the RCA are shown, demonstrating the intramural portion (c) and just distally (d).

**Figure 5:**
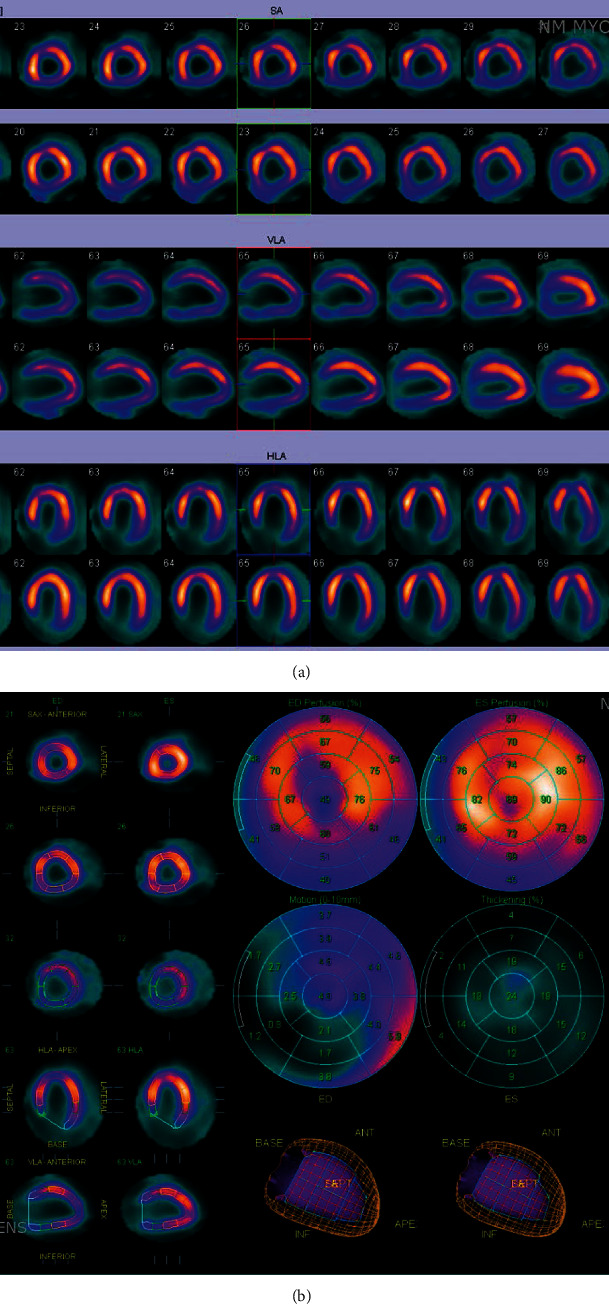
(a, b) The patient achieved 11 METS on a modified Bruce protocol but developed symptomatic chest discomfort and inferior lead ST-segment depressions at peak exercise persisting 1 minute into recovery. SPECT images demonstrated a reversible inferior wall perfusion deficit.

Transthoracic echocardiogram (TTE) showed severe left ventricular dilatation (end-diastolic diameter of 6.6 cm) with normal wall thickness and ejection fraction (LVEF) of 30% with basal inferior and inferolateral akinesis. Coronary angiography showed mild nonobstructive coronary artery disease and an anomalous right coronary artery (ARCA) arising from the left coronary cusp (Figure 1 and Video 1). Iron and thyroid indices, hepatitis serologies, and antibodies against HIV and *Trypanosoma cruzi* were unremarkable. Cardiac magnetic resonance imaging (CMR) was obtained given suspicion for myocarditis as a cause of dilated cardiomyopathy which corroborated severe left ventricular dysfunction with ejection fraction of 16% (Video 2), but no myocardial edema on T2-weighted images or late gadolinium enhancement (LGE) (Figures 2(a) and 2(b)). Axial scout images again demonstrated an ARCA arising from the left coronary cusp, but now clearly appearing to take an intra-arterial course between the aorta and the main pulmonary artery (Figure 3). Cardiac computed tomographic angiography (CCTA) was obtained to better delineate its course, confirming a slit-like proximal orifice arising at an acute angle and an intramural and intra-arterial course between the great vessels (Figures 4(a)–4(d)). Exercise single-photon emission computed tomography (SPECT) demonstrated a mild-to-moderate-sized reversible defect in the inferolateral wall referable to the territory supplied by the ARCA (Figures 5(a) and 5(b)).

## 3. Management

The patient's presenting symptoms eventually improved with a medical regimen including a diuretic, beta-blocker, ACE-inhibitor, and aldosterone antagonist. His chest pain, biomarker elevation, and inducible ischemia on SPECT were hypothesized as consequential to his ARCA, albeit disproportionate to the degree of left ventricular dysfunction, which was ascribed to a more global, nonischemic cardiomyopathic process. Given the presence of inducible ischemia in the RCA territory, there was a consensus to proceed with surgical unroofing of the right coronary ostium. He tolerated the operation well and was discharged on postoperative day 7 on a guideline-directed regimen for heart failure.

## 4. Follow-Up

He was seen in a follow-up three weeks after discharge with complete resolution of his exertional chest pain and shortness of breath. He returned back to work and remained asymptomatic.

## 5. Discussion

Congenital coronary anomalies (CA) refer to a family of aberrations including anomalies of the origin, course and number of epicardial coronary arteries [1]. They have an estimated prevalence of 0.2-1.0%, with a slight male predilection reported in observational series [2]. ARCA arising from the left sinus of Valsalva is more common than anomalous left coronary artery (ALCA) from the right sinus of Valsalva [3]. CA are usually diagnosed on the basis of their presenting symptoms or incidentally among patients undergoing coronary angiography, CCTA, or at autopsy [4]. Whereas most CA are asymptomatic, presentation with arrhythmia, syncope, myocardial infarction, or sudden death has been described. For patients who present with sudden death, mean age of presentation is reported in various studies to range from teenage years to the fourth decade [5]. Patients who present with ischemia generally do so before the fifth decade, and the degree of troponin elevation is highly variable [6]. Systolic dysfunction and heart failure are rare consequences of CA, with most reported cases associated with anomalous left coronary artery arising from the pulmonary artery (ALCAPA) [7]. To the best of our knowledge, this case is the first report of systolic dysfunction and dilated cardiomyopathy associated with ARCA.

Our patient presented with angina, signs and symptoms of heart failure, and profound systolic dysfunction with inferior ischemia on SPECT imaging. Following an initial ischemic evaluation with coronary angiography (which first disclosed the ARCA), his degree of the ventricular dysfunction, coupled with the biomarker trend, prompted a diagnostic workup for myocarditis with CMR. CCTA was subsequently needed to delineate the distal course of the ARCA and to identify possible high-risk features. Finally, SPECT perfusion imaging was needed to identify inducible ischemia.

A multimodal imaging approach as exemplified in this case is often required to comprehensively risk stratify patients with CA. In our patient, ARCA was first detected incidentally via coronary angiography, which is now less commonly utilized as a first-line modality due to its invasive nature and limited ability to assess the full course of the vessel and surrounding noncoronary anatomy [8]. CCTA has thus emerged as the first-line imaging modality to assess the origin and course of CA [9]. This is owing to its high spatial resolution and ability to identify high-risk features including a slit-like orifice, acute angle takeoff, or intramural course [10]. CMR is another noninvasive method that has the added benefit of being able to assess cardiac function and areas of myocardial fibrosis, but is limited in terms of its spatial resolution and ability to track the arterial course beyond the proximal aspect [11].

The functional relevance of any detected CA, especially when incidental, often requires further clarification with myocardial perfusion imaging. This can be done using SPECT, positron emission tomography (PET), or CMR. Notable anatomic factors associated with high risk for ischemia on perfusion imaging include an intramural and intra-arterial course between the great vessels, acute angulation at the ostium, and a slit-like orifice, all features which were present in our patient [12].

Management strategies for CA include observation, medical therapy with beta-blockers, stenting, and surgical unroofing [13]. In cases of ARCA with evidence of ischemia, surgical revascularization is a class I recommendation per the joint guidelines of the American College of Cardiology and American Heart Association [14].

## 6. Conclusions

ARCA is a well known cause of myocardial ischemia. A multimodal imaging approach is often required in order to rule out concomitant diagnoses and to definitively delineate the anomalous course. Surgical correction should be pursued in symptomatic patients or in those with evidence of myocardial ischemia attributed to the anomalous coronary artery.

## Data Availability

All data used in this case report is readily available through cited literature or is protected patient information which cannot be released.

## Abbreviations

TTE: Transthoracic echocardiography
CMR: Cardiac magnetic resonance
CCTA: Coronary computed tomographic angiography
SPECT: Single-photon emission computed tomography
CA: Coronary anomalies
ARCA: Anomalous right coronary artery
ALCA: Anomalous left coronary artery
LGE: Late gadolinium enhancement
LVEF: Left ventricular ejection fraction
MPI: Myocardial perfusion imaging.

## Supplementary Materials

Supplementary 1 Video 1: cine-angiographic images demonstrating the anomalous RCA in the RAO projection.

Supplementary 2 Video 2: steady-state free precession cine images in the four-chamber view demonstrating severely reduced biventricular systolic function.
